# Severe Acute Hepatitis in a COVID‐19 patient: A Case Report

**DOI:** 10.1002/ccr3.4869

**Published:** 2021-10-13

**Authors:** Shakiba Dehghani, Azam Teimouri

**Affiliations:** ^1^ School of medicine Isfahan University of Medical Sciences Isfahan Iran; ^2^ Isfahan Gastroenterology and Hepatology Research Center Isfahan University of medical sciences Isfahan Iran; ^3^ Metabolic Liver Disease Research Center Isfahan University of Medical Science Isfahan Iran

**Keywords:** acute hepatitis, COVID‐19, hepatitis A, herpes simplex virus

## Abstract

Liver enzymes abnormalities are one of the reported presentations of coronavirus infection mostly in hospitalized patients. It is important that physicians take all the possible causes of acute hepatitis in consideration when dealing with abnormal liver enzymes in a patient with COVID‐19 infection to reduce the risk of overlooking the underlying disease. Hereby, we reported case of a 39‐year‐Old man who presented with severe acute hepatitis and was infected with COVID‐19, hepatitis A and herpes simplex virus simultaneously.

## Graphical abstract

Liver enzyme abnormalities can be a COVID‐19 infection manifestation but physicians should consider other causes of acute hepatitis when dealing with elevated liver enzymes in a COVID‐19 patient.

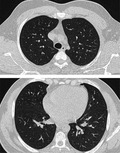

## 1 INTRODUCTION

Coronavirus (COVID‐19), caused by the novel coronavirus SARS‐CoV‐2, was first reported in December 2019 in China and has since spread widely throughout the world, affecting over 180 million patients and resulting in over 4 million deaths as of the time of writing this article. In 2020, it was declared a global public health emergency.^1^, ^2^

Even though numerous studies have been conducted since its emergence, many aspects of this virus remain unknown. Clinical manifestations vary considerably between individuals and affect a variety of organs.^3^

Co‐infections with COVID‐19 are not uncommon. Because the presentation of COVID‐19 can be similar to that of many other infectious diseases, misdiagnosis or delayed diagnosis is unavoidable, and in some cases, can result in severe consequences.^4^ Thus, it appears critical to report other concurrent infectious diseases associated with COVID‐19 infection to inform clinicians about the dangers of overlooking coexisting diseases.

In this paper, the case of a 39‐year‐old man is presented who was diagnosed with hepatitis A, herpes simplex virus, and COVID‐19 infection concurrently.

## 2 CASE PRESENTATION

**FIGURE 1:**
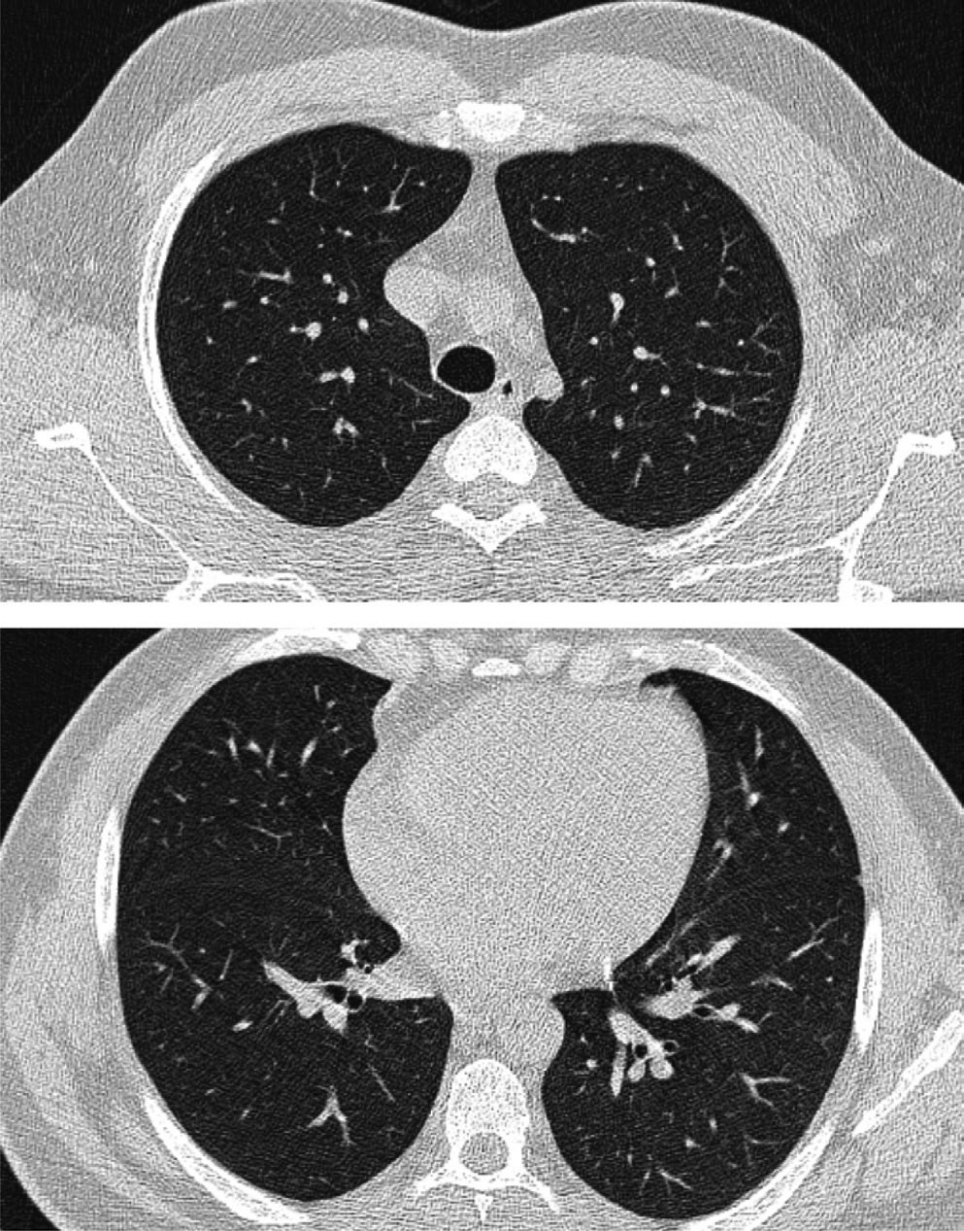
Two axial cuts of lung HRCT of the patient on day first of admission

A 39‐year‐old man presented with severe myalgia, refusal to eat, headache, fever, fatigue, and weakness over a two‐day period. He stated that he had no respiratory symptoms such as chest pain, coughing, sore throat and was not nauseated, vomiting, experiencing diarrhea, or abdominal pain. He also stated that he had no prior underlying conditions. His vital signs were stable during the physical examination, and his oxygen saturation was 96% on room air. A nasopharyngeal swab was performed promptly, and an RT‐PCR confirmed COVID‐19 infection. A lung CT scan was then performed, which revealed no abnormalities. (Figure 1) He was then treated in an outpatient setting for symptomatic management and supportive care.

He was referred to our hospital a few days later with jaundice, recent darkening of urine, and lightening of stool. His vital signs were stable at the time. His body temperature was 37℃, and his oxygen saturation was 95% on room air. His sclera and skin were unambiguously icteric. No cutaneous manifestations were observed, and cardiac and pulmonary examinations were normal. The abdominal examination revealed hepatomegaly with a liver span of approximately 16 centimeters but no tenderness in the right upper quadrant. Physical examination revealed no additional positive findings.

Additional history taking was conducted regarding jaundice. The patient made no complaints of itchiness, abdominal pain, recent changes in mental status, or insomnia. He disclosed that he had recently camped and drank water from natural springs. He also stated that he was not taking any medication, including herbal compounds, drinking alcohol or injecting drugs, engaging in unprotected sexual activity, or recently being exposed to a jaundiced person. He was not related to anyone with a history of liver disease. Then, preliminary blood tests were performed to determine the presence of jaundice. The following were the findings: serum alanine aminotransferase (ALT) 2228 IU/L, serum aspartate aminotransferase (AST) 1241 IU/L, alkaline phosphatase (ALP) 410, INR 1.4, Albumin 3.4 g/dL, serum total bilirubin 11.2 mg/dL, and serum direct bilirubin 7.2 mg/dL.

He was initially diagnosed with severe acute hepatitis and admitted. Due to the progressive pattern of abnormal liver function and the risk of developing acute liver failure, a liver transplant center was consulted, but he was deemed unsuitable for transplantation due to his infection with COVID‐19.

Although elevated liver enzymes are a common manifestation of COVID‐19 infection, we performed a thorough work‐up to rule out other possible causes of abnormal liver enzymes. Meanwhile, AST and ALT levels rapidly decreased to 186 and 564 IU/L, respectively, but ALP, total and direct bilirubin, and INR levels increased to 663 IU/L, 13.2 mg/dL, 8.4 mg/dL, and 1.64, respectively, on day three of admission. No new symptoms, such as abdominal pain or tenderness, had developed in the patient. An abdominal ultrasound was performed to rule out the presence of cholestasis. Due to the normal ultrasound and the continuing rise in ALP, INR, and bilirubin levels, an MRCP was performed to rule out cholestatic causes. Hepatitis A virus (HAV) antibody IgM and Herpes simplex virus (HSV) were positive in establishing the hepatocellular cause of elevated liver aminotransferase.

Because liver enzymes decreased rapidly while receiving only supportive care, only symptomatic and supportive care was continued, and no other treatment was initiated. ALP, bilirubin, and INR levels all decreased during follow‐up, in addition to ALT and AST levels. All liver chemistry studies improved and returned to normal within two months of presentation.

## 3 DISCUSSION

COVID‐19 infection manifests itself in various ways, including respiratory symptoms (the most prevalent), cardiac involvement, neurological symptoms, kidney injury, and gastrointestinal symptoms.^3^

Another frequently reported symptom of COVID‐19 infection is abnormal liver enzyme levels. This symptom has been observed in up to 76% of COVID‐19 patients.^5^ The majority of reported cases had only a mild elevation of liver enzymes (<2 times ULN), and only 6% of reported cases had severe liver enzyme changes (>5 times ULN).^6^ This symptom is most frequently observed in hospitalized patients infected with COVID‐19, but liver enzyme abnormalities have been reported in the absence of respiratory symptoms in subjects infected with COVID‐19 on rare occasions.^7^

Although several pathways have been proposed to be associated with changes in liver enzymes, the exact cause is unknown. The mechanisms postulated include cholangiocyte damage caused by the virus directly binding to angiotensin‐converting enzyme 2, hepatic congestion, ischemic hepatitis, cell‐mediated apoptosis, venous and arterial thromboses, drugs, and extrahepatic causes of transaminase release such as myositis.^8^, ^9^

Hepatitis A virus (HAV) is an RNA virus transmitted via fecal‐oral routes and causes fatigue, weakness, malaise, fever, and jaundice. Although ALT and AST levels can exceed 1000 U/dL in hepatitis A, total bilirubin levels rarely exceed 10 mg/dL. Acute hepatitis A infection is diagnosed by the presence of anti‐HAV IgM in the serum. There are no specific treatments for Hepatitis A infection, and symptomatic patients should receive supportive care.^10^

Herpes simplex virus (HSV) is a DNA virus that causes acute viral hepatitis in a rare number of cases. Clinically, it is indistinguishable from other causes of acute viral hepatitis, and it is frequently associated with a poor prognosis due to delayed diagnosis and treatment.^11^

Although there have been reports of COVID‐19 co‐infection with HSV, no cases of COVID‐19 infection with hepatitis A or HSV have been reported to our knowledge.

In this paper, a case was presented of a patient who was simultaneously infected with three viruses.

COVID‐19 infection, on the one hand, and concurrent HAV and HSV infections, on the other hand, aggravated liver function in this patient, resulting in severe acute hepatitis. Only two months after the onset of symptoms, all liver chemistries, and coagulation studies returned to normal ranges with supportive treatment.

In conclusion, physicians should consider all possible causes of acute hepatitis when a patient with COVID‐19 infection has abnormal liver enzymes, and not all changes should be attributed to COVID‐19 infection. Making the correct diagnosis at the appropriate time can significantly affect how patients and the underlying disease are managed.

## CONFLICTS OF INTEREST

The authors declare that they have no competing interests.

## AUTHOR CONTRIBUTIONS

Shakiba Dehghani had contributed in conducting the study and manuscript preparation. Azam Teimouri had contributed in designing and conducting the study. All authors have revised the manuscript critically for important intellectual content, also have read and approved the content of the manuscript and confirmed the accuracy or integrity of any part of the work.

## ETHICAL APPROVAL

This report has been performed in accordance with the Declaration of Helsinki.

## Data Availability

The datasets used during the current study are available from the corresponding author on request.
